# A bibliometric and visualized analysis of meniscus suture based on the WOS core collection from 2010 to 2022: A review

**DOI:** 10.1097/MD.0000000000034995

**Published:** 2023-11-17

**Authors:** Xu Liu, Di Liu, Michael Opoku, Wenhao Lu, Linyuan Pan, Yusheng Li, Heyuan Zhu, Wenfeng Xiao

**Affiliations:** a Department of Orthopedics, Xiangya Hospital, Central South University, Changsha, Hunan, China; b National Clinical Research Center for Geriatric Disorders, Xiangya Hospital, Central South University, Changsha, Hunan, China; c Department of Orthopedics, Central hospital of Loudi, Loudi, Hunan, China.

**Keywords:** bibliometric analysis, hotspots, meniscus suture, research trend, suture technique

## Abstract

Meniscus suture is an important treatment method for meniscus injury and contributes to the preservation of proprioception, restoration of knee biomechanics and alleviation of progressive osteoarthritis. However, there are few visualized analyses concerning the present studies of meniscus suture. This paper aims to evaluate the global trends, highlights and frontiers of meniscus suture. A bibliometric analysis was conducted based on the results of studies related to meniscus suture from web of science core collection. VOSviewer, GraphPad Prism, Microsoft Excel and R-bibliometrix were utilized for the bibliometric analysis of country and institution distribution, chronological distribution, source journals analysis, prolific authors and institutions analysis, keywords analysis, and reference co-citation analysis. A total of 950 publications on meniscus suture from 177 different sources were retrieved over the set time span. These publications were completed by 3177 authors from 1112 institutions in 54 countries. The United States was the most prolific country with 7960 citations and 348 publications (36.63%). Furumatsu Takayuki acted as the most prolific author (51 publications), while Robert F LaPrade with 1398 citations was the most-cited author. And more papers were published in the core journals, including *American Journal of Sports Medicine, Arthroscopy-The Journal of Arthroscopic and Related Surgery, Knee Surgery Sports Traumatology Arthroscopy* and *Arthroscopy Techniques*. Furthermore, “meniscus healing,” “meniscus root tear” seem to be the emerging research hotspots. Notably, the publication trend concerning the all-inside suture technique has been rising during the past decade. The number of research publications on meniscus suture has been continuously risen since 2010. The authors, publications and institutions from the United States and East Asia were still the mainstays in this field. And the all-inside suture may become the mainstream surgical technique in the future, with meniscus healing and meniscus root tears being research highlights recently.

## 1. Introduction

As a normal structure in the knee joint, meniscus plays an important role in load distribution, shock absorption, stability, nutrition, lubrication and proprioception.^[1]^ Meniscus tear is a common cause of knee pain and mobility impairment, with an annual average incidence of 61 to 70 per 100,000 in the United States, especially in the young and physically active population.^[2,3]^ Moreover, meniscal injury caused heavy social-economic burden per year.^[4,5]^ In clinical practice, meniscus tears were generally divided into traumatic tears and degenerative tears. Traumatic tears mainly appeared in young people mainly due to knee injury, while degenerative tears are mainly prevalent in elderly patients related to repeated wear of meniscus or chondromalacia.^[6,7]^ Furthermore, increased activity level, body mass index, age, gender, and anatomy were considered as the important risk factors.^[2]^

Surgical treatment remains the primary option for traumatic meniscus tears, especially in unstable traumatic meniscus tears.^[8]^ With more than 700,000 cases in the United States per year, arthroscopy partial meniscectomy as the gold standard, is the most commonly performed orthopedic procedure.^[9]^ However, an increasing number of evidences has demonstrated that removing meniscus increases the incidence of knee osteoarthritis (KOA).^[10–12]^ Consequently, with the development of arthroscopic techniques, the meniscus suture has gradually emerged the primary surgical strategy during the past decade.^[13–17]^ Meniscus suture contained various surgical techniques: inside-out suture, outside-in suture, and all-inside suture.^[18]^ In addition, compared to arthroscopy partial meniscectomy, meniscus suture reduced the incidence of KOA with a higher level of activity and patient satisfaction in the long term.^[8,19–21]^ Meniscus suture is still in progress yet, as multiple factors can influence the success of meniscus suturing (joint stability, rehabilitation strategies, age, tear type, heal stimulation).^[22–24]^ Therefore, further research is needed.

Bibliometrics analysis can be used to quantify the impact of individual research results and the development of literature on specific topics, assess research hotspots and predict future research directions in the growing number of papers.^[25,26]^ At present, complete procedures and research tools for bibliometrics were available and it has been applied in different fields.^[27,28]^ The web of science core collection (WOSCC) as the data source and using VOSviewer and R-bibliometrix together were widely recommended.^[28–31]^ In this study, we analyzed the recently published studies concerning the meniscus suture by using bibliometrics analysis and provided valuable illuminations for future research in this field.

## 2. Materials and methods

### 2.1. Data source and search strategy

We conducted a literature search on meniscus suture in the WOSCC on January 10, 2023. The publication year was set as 2010 to 2022, and the search formula was as follows: (TI = [Meniscus OR Menisci OR Meniscal] AND TI = [suture OR suturing OR repair]) AND PY = [2010–2022]). To avoid deviations due to data updates, the above operations were completed on January 10, 2023.

### 2.2. Data extraction and statistical analysis

Two different researchers completed the search separately to avoid duplicate publications and negative results, and all the retrieved articles were used in the bibliometric analysis.

VOSviewer (version 1.6.18) was used to build bibliometric networks, to identify productive institutions, authors, citations, and to construct related visual networks. In addition, R-bibliometrix was used through RStudio for further data analysis and visual presentation of the search results. Moreover, Microsoft Excel 2019, used for manual verification to avoid errors caused by software and to calculate the frequency and percentage of published material.

### 2.3. Relevant bibliometric indicators

We respectively used the H-index, M-index, dominance factor (DF), and G-index to assess the academic influence of an author.

1. H-index: If each of the H papers published by the author has been cited at least H times, the value H is called the H index of the author.
2. M-index: M-index is the H-index divided by the author academic age, which refers to the time since the scholar published his first paper to this field.

$$\begin{array}{l} M-index=\frac{H-index}{Y_{academic\;age}} \end{array}$$

M-index can exclude the influence of academic age when we evaluate authors.

1. G-index: If a scientist has published at least N articles, and the number of citations of these N articles is accumulated at least N² times, N is the G-index of the scientist.

G-index is more likely to reflect the academic achievements of the scientists with fewer publications but higher citations.

1. DF: DF is the frequency with which an author appears as the first author in all coauthored publications.

$$\begin{array}{l} DF=\frac{P_{first}}{P_{coauthor}} \end{array}$$

High DF value shows more dominance of author as first author.

## 3. Results

### 3.1. Search results description

A total of 950 publications were retrieved from the WOS core collection as showed in Table 1. In the search results, article (736, 77.47%) accounted for the majority, followed by reviews (101, 10.63%), editorial materials (38, 4.00%), meeting abstract (36, 3.79%) and letter (24, 2.52%). The proportion of proceeding paper, correction and news item is all <1%.

**Table 1:** Types of retrieved documents.

Documents type	Total publications (TP)	Percentage (%)
Article	736	77.47
Proceedings paper	8	0.84
Correction	6	0.63
Editorial material	38	4.00
Letter	24	2.52
Meeting abstract	36	3.79
News item	1	0.10
Review	101	10.63
Total	950	100.00

### 3.2. Publications growth trend and citations

**Figure 1.**
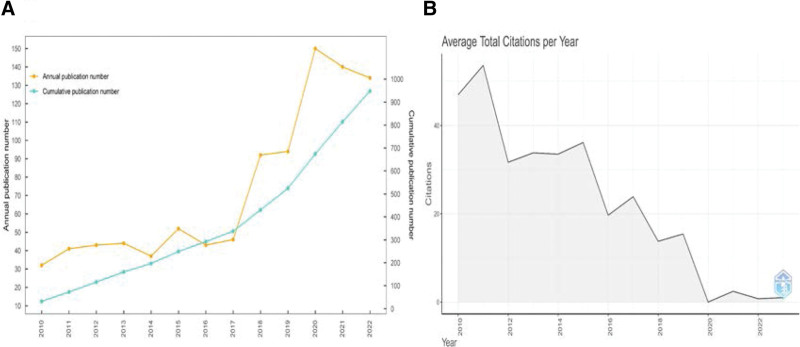
(A) Visualization of the annual publication number and the cumulative publication number. (B) Average total citations per y since 2010.

There was an increasing trend in the number of articles on meniscus suture from 2010 to 2022. Table 2 shows the number of published papers and citations for all the years, with the highest number of papers published in 2020 reaching 150. Figure 1A shows the annual and cumulative number of publications on meniscus suturing from 2010 to 2022 and there was an apparent growth of papers in 2018 and 2020. The average total citations in each year were shown in Figure 1B, which had a downward trend, and 2011 had the highest average number of citations.

**Table 2:** Annual number of publications and citations.

Y	Articles	Total citations (TC)	Average citation
2010	32	1582	49.44
2011	41	2317	56.51
2012	43	1447	33.65
2013	44	1556	35.36
2014	37	1302	35.19
2015	52	1930	37.12
2016	43	874	20.34
2017	46	1124	24.43
2018	92	1325	14.40
2019	94	1505	16.01
2020	150	933	6.22
2021	140	347	2.48
2022	136	97	0.71

### 3.3. The distribution of nations and institutions

**Figure 2.**
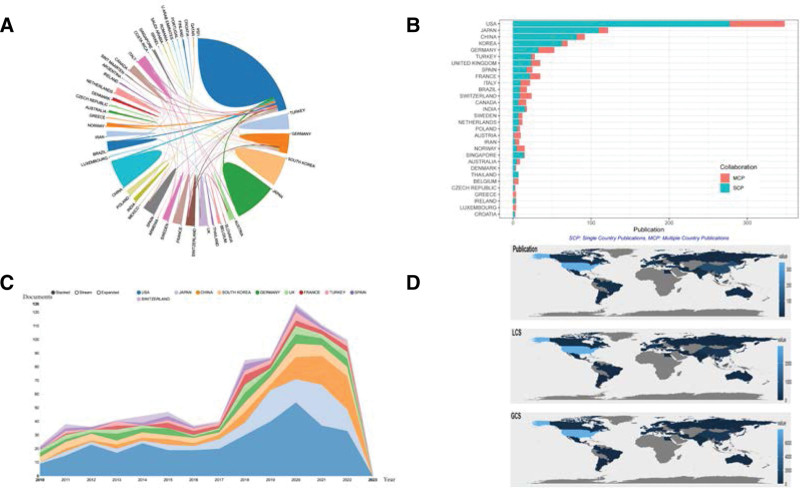
(A) A visual network of national cooperation. (B) Distribution of publications produced by single country or by multiple countries. (C) Annual number of publications in the top 10 countries. (D) Geographic distribution of national publications, LCS and GCS. A and C were made in an online analysis platform (https://bibliometric.com). GCS = global citation score, LCS = local citation score.

A total of 950 articles were retrieved from 54 countries and regions, most of which were from East Asia and Europe, including the United States. The top 10 countries by publications or citations are shown in Table 3: the United States ranked first with 348 publications, and Japan, China, South Korea and Germany followed with 122, 92, 70, and 53, respectively. The United States also ranked first in citations with 7963 times, and the gap between USA with other countries is huge. Figure 2A shows the national cooperation network. The United States is an important contributor to meniscus suture research and has close cooperation with Brazil, Japan, and China, but the cooperation between other countries is not frequent. Figure 2B also shows that the USA has a higher proportion of multi-country publications than other countries. A transformative trend of annual publications of the top 10 countries/regions is shown in Figure 2C. The United States published the most documents, and China, Japan and South Korea published a great increase in the past 5 years. The geographical distribution of global publications and of the number of local citation score and global citation score (GCS) are shown in Figure 2D. Most countries except Africa have published the relevant papers, and the United States has the most publications, local citation score and GCS.

**Table 3:** The top 10 countries/regions by publications, citations.

Rank	Country/region	Publications	Percentage (%)	Rank	Country/Region	Total citations	Average citations
1	USA	348	36.63	1	USA	7963	22.88
2	Japan	122	11.79	2	Korea	1744	24.91
3	China	92	9.68	3	Japan	1380	11.31
4	Korea	70	7.37	4	Germany	1298	24.49
5	Germany	53	5.58	5	China	789	8.58
6	France	35	3.68	6	France	705	20.14
7	UK	35	3.68	7	UK	700	20.00
8	Turkey	28	2.95	8	Italy	429	19.50
9	Spain	25	2.63	9	Spain	387	15.48
10	Switzerland	24	2.53	10	Canada	371	21.82

**Figure 3.**
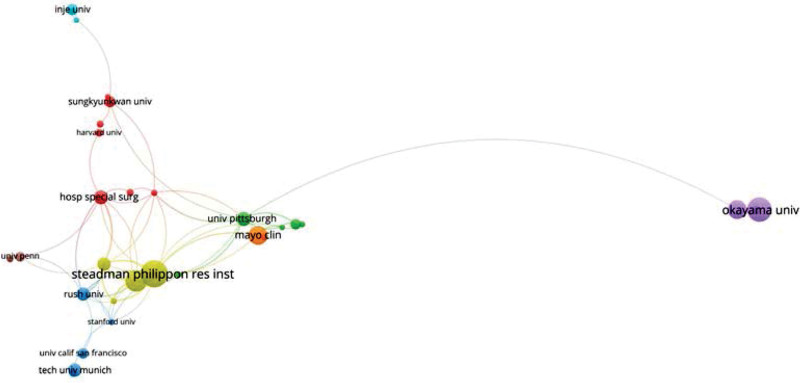
A visual network of institutions collaboration (T = 5).

Figure 3 is the visualization network of institutional cooperation. The inclusion criteria are institutions that have published at least 5 papers (T = 5). The Steadman Clinic and Steadman Philippon Research institution from the United States have the larger nodes, meaning they have more publications than other institutions. Furthermore, those 2 institutions frequently cooperate with other institutions, such as Rush University, Hospital for Special Surgery and Mayo Clinic.

### 3.4. Keywords analysis

**Figure 4.**
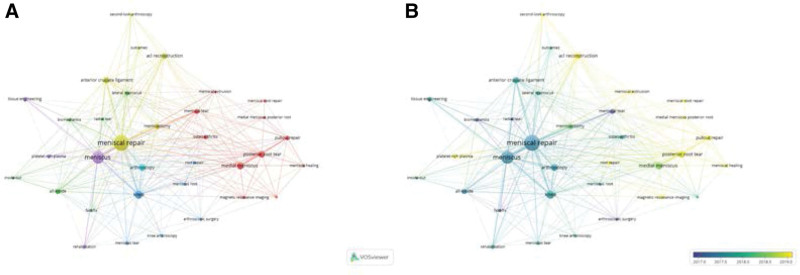
(A) The network visual map of author keywords. (B) The network visual map of author keywords based on time span (2017–2019). Yellow indicates the terms that appeared later. Both (A) and (B) were made by VOSviewer.

In total of 950 documents, 1129 Author keywords (DE) and 957 Author keywords-plus (ID) were extracted by using RStudio. Figure 4A is a visual network using VOSviewer for Author keywords that appear more than 10 times. There are 5 main distinct colored clusters that appear in Figure 4A, and each cluster represents some aspects of the research in meniscal suture. Red cluster (root tears study), yellow cluster (combined injury study), blue cluster (arthroscopy study), green cluster (suture technique study), and purple cluster (rehabilitation study). As can be seen from Figure 4B, research hotspots in recent years mainly focus on “meniscus healing” and “meniscus root tear.”

### 3.5. Classification of journals

**Figure 5.**
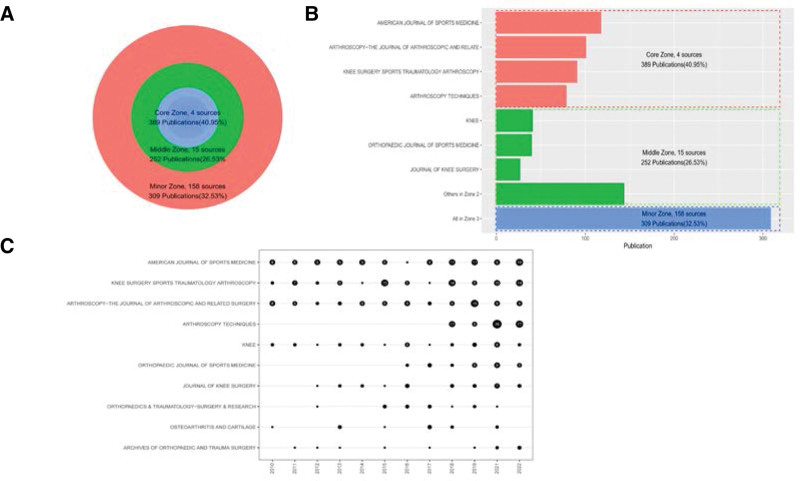
Visualization of important journals. (A) The 177 journals are divided into 3 zones. (B) Publication volume of several important journals in the 3 zones. (C) The number of annual articles published by the top ten most prolific journals.

All 950 articles were published in journals, and there were 177 different journals retrieved. According to Bradford law, we artificially divided the 177 journals into 3 zones: core zone (4 journals), middle zone (15 journals), and minor zone (158 journals). The journals from core zone have published 389 documents (40.95%), and middle zone (252 documents, 26.53%), minor zone (309 documents, 32.53%), as shown in Figure 5A. Table 4 lists the top 10 journals by number of publications, including the JIF quartile and impact factor of each journal. The core zone journals were composed by *American Journal of Sports Medicine, Arthroscopy-The Journal of Arthroscopic and Related Surgery, Knee Surgery Sports Traumatology Arthroscopy* and *Arthroscopy Techniques*. Except *Arthroscopy Techniques*, the other 3 journals belong to Q1. The publications of some important journals in the 3 zones are visualized in Figure 5B, easily seeing that the core zone journals published more documents than other journals. The annual number of articles in top 10 journals is shown in Figure 5C, finding that *Arthroscopy Techniques* did not publish any paper until 2018, but the number of publications from *Arthroscopy Techniques* in each year was large.

**Table 4:** The top 10 journals by number of publications.

Rank	Journal	Articles	Percentage (%)	JIF quartile	IF (JCR 2021)
1	AMERICAN JOURNAL OF SPORTS MEDICINE	118	12.42	Q1	7.01
2	ARTHROSCOPY-THE JOURNAL OF ARTHROSCOPIC AND RELATED SURGERY	101	10.63	Q1	5.973
3	KNEE SURGERY SPORTS TRAUMATOLOGY ARTHROSCOPY	91	9.58	Q1	4.114
4	ARTHROSCOPY TECHNIQUES	79	8.32	NA	NA
5	KNEE	41	4.32	Q3	2.423
6	ORTHOPAEDIC JOURNAL OF SPORTS MEDICINE	40	4.21	Q2	3.401
7	JOURNAL OF KNEE SURGERY	27	2.84	Q3	2.501
8	ARCHIVES OF ORTHOPAEDIC AND TRAUMA SURGERY	17	1.79	Q2	2.928
9	ORTHOPAEDICS & TRAUMATOLOGY-SURGERY & RESEARCH	16	1.68	Q3	2.425
10	OSTEOARTHRITIS AND CARTILAGE	16	1.68	Q1	7.507

### 3.6. Analysis of important publications

The citation times of each publication were calculated by RStudio, and the top ten publications with total citation times were listed in Table 5. As shown in Figure 1B, the most average citation was in 2011, and the top 2 cited-articles in Table 5 were both published in 2011.^[12,32]^ The first publication in Table 5 was a review done by Eleftherios A Makris and his colleagues in 2011, summarizing the structure and function of meniscus, pathophysiology of meniscus and tissue engineering of meniscus.^[32]^ In addition, a systematic review conducted by Paxton E Scott al et. was the second most cited production.^[12]^ This review compared the outcomes between meniscectomy and arthroscopic meniscal suture (inside-out, outside-in and all-inside). And it indicated that meniscus sutures have a higher reoperation rate than partial meniscectomies, but meniscus sutures seemed to have better long-term outcomes.^[12]^

**Table 5:** Top 10 publications by citations.

Rank	Title	Author	GCS	LCS	DOI	Y
1	The knee meniscus: Structure-function, pathophysiology, current repair techniques, and prospects for regeneration	Makris, Eleftherios A.	553	68	10.1016/j.biomaterials.2011.06.037	2011
2	Meniscal Repair Versus Partial Meniscectomy: A Systematic Review Comparing Reoperation Rates and Clinical Outcomes	Paxton, E. Scott	269	107	10.1016/j.arthro.2011.03.088	2011
3	Long-Term Outcome After Arthroscopic Meniscal Repair Versus Arthroscopic Partial Meniscectomy for Traumatic Meniscal Tears	Stein, Thomas	261	117	10.1177/0363546510364052	2010
4	Dynamic Contact Mechanics of the Medial Trends in Meniscus Repair and Meniscectomy in the United States, 2005–2011	Abrams, Geoffrey D.	231	75	10.1177/0363546513495641	2013
5	Surgical and tissue engineering strategies for articular cartilage and meniscus repair	Kwon, Heenam	230	6	10.1038/s41584-019-0255-1	2019
6	Biomechanical Consequences of a Complete Dynamic Contact Mechanics of the Medial Meniscus as a Function of Radial Tear, Repair, and Partial Meniscectomy	Bedi, Asheesh	196	45	10.2106/JBJS.I.00539	2010
7	Altered Tibiofemoral Contact Mechanics Due to Meniscal Repair Outcomes at Greater Than 5 y A Systematic Literature Review and Meta-Analysis	Nepple, Jeffrey J.	196	107	10.2106/JBJS.K.01584	2012
8	Biomechanical Consequences of a Complete Radial Tear Adjacent to the Medial Meniscus Posterior Root Attachment Site In Situ Pull-out Repair Restores Derangement of Joint Mechanics	Padalecki, Jeffrey R.	169	89	10.1177/0363546513499314	2014
9	Altered Tibiofemoral Contact Mechanics Due to Lateral Meniscus Posterior Horn Root Avulsions and Radial Tears Can Be Restored with in Situ Pull-Out Suture Repairs	LaPrade, Christopher M.	164	59	10.2106/JBJS.L.01252	2014
10	Arthroscopic Suture Anchor Repair Versus Pullout Suture Repair in Posterior Root Tear of the Medial Meniscus: A Prospective Comparison Study	Kim, Jae-Hwa	141	82	10.1016/j.arthro.2011.06.033	2011

GCS = global citation score, LCS = local citation score.

### 3.7. Co-cited references analysis

**Figure 6.**
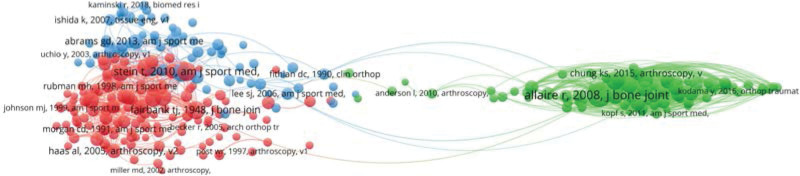
Network visualization of important authors collaboration. The minimum number of articles of an author is 5.

**Figure 7.**
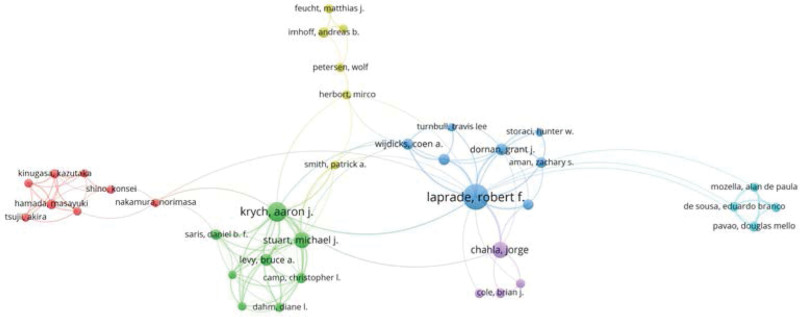
A visual network map of co-cited references (T = 20) made by VOSviewer.

A total of 9860 references appeared in all 950 publications retrieved and the top ten references with co-occurrence times were shown in Table 6. References with more than 20 co-citations (T = 20) were extracted and used to form the co-citation map (Fig. 6), which included 273 references. The red and blue clusters were more closely related than the green clusters, meaning that references in these 2 clusters were more frequently cited together. The references “R. Allaire 2008, j bone joint,”^[13]^ “Stein T, 2010, am j sport med,”^[11]^ “Fairbank TJ, 1948, j bone joint”^[33]^ were more frequently co-cited (Fig. 7). The paper written by Fairbank from 1948 was earlier to research the relation between meniscectomy and osteoarthritis.^[33]^ A cadaver experiment successfully demonstrated that the posterior root tear led to bad biomechanical consequences, greatly promoting the research of meniscus root tears.^[13]^ The study conducted by Thomas Stein firstly estimated the osteoarthritic progress, function and sports activity in a long-term comparison between arthroscopic meniscal repair and partial meniscectomy, and the positive outcomes of meniscus repair stimulated the further studies.^[11]^

**Table 6:** The top 10 co-cited references.

Rank	Co-cited reference	Count
1	Biomechanical consequences of a tear of the posterior root of the medial meniscus	188
2	Long-term outcome after arthroscopic meniscal repair versus arthroscopic partial meniscectomy for traumatic meniscal tears	177
3	Knee joint changes after meniscectomy	110
4	Meniscal Root Tears Significance, Diagnosis, and Treatment	110
5	Meniscal repair versus partial meniscectomy: a systematic review comparing reoperation rates and clinical outcomes	107
6	Meniscal repair outcomes at greater than 5 y: a systematic literature review and meta-analysis	107
7	Microvasculature of the human meniscus	105
8	Meniscal tears: the effect of meniscectomy and of repair on intraarticular contact areas and stress in the human knee. A preliminary report	90
9	Biomechanical consequences of a complete radial tear adjacent to the medial meniscus posterior root attachment site: in situ pull-out repair restores derangement of joint mechanics	89
10	Comparison of inside-out and all-inside techniques for the repair of isolated meniscal tears: a systematic review	88

### 3.8. Analysis of prolific authors

A total of 3177 authors contributed to the 950 retrieved articles, including 46 single-author articles, and coauthors documents had an average of 5.50 authors. Furumatsu Takayuki was the author with the most publications and Robert F Laprade had the highest number of citations with highest H-index and G-index. Okazaki, Y, who was the first author of 14 publications, reached the first place with a DF of 0.341 (Table 7). Robert F Laprade and his group members at the Steadman Philippon Research Institution have worked closely together and contributed greatly to the field of meniscus suture. We also found that Aaron J Krych and his team from Mayo Clinic also made a great contribution (Fig. 6).

**Table 7:** The top 10 most prolific authors in meniscus suture research.

Rank	Authors	Publications	Total Citations	Average citations	H-index	G-index	M-index	Publications (first author)	DF	Country/region
1	Furumatsu, T	51	456	8.94	14	4	1.56	9	0.176	Japan
2	Laprade, RF	48	1398	29.13	20	7	1.67	5	0.104	USA
3	Ozaki, T	48	455	9.48	14	4	1.56	0	0	Japan
4	Okazaki, Y	41	283	6.90	11	4	2.20	14	0.341	Japan
5	Hiranaka, T	38	220	5.79	10	4	2.50	12	0.316	Japan
6	Kamatsuki, Y	37	319	8.59	12	4	2.00	3	0.081	Japan
7	Kodama, Y	36	379	10.53	12	4	1.71	2	0.056	Japan
8	Krych, AJ	27	431	15.96	12	4	0.92	6	0.222	USA
9	Miyazawa, S	22	304	13.82	12	4	1.33	0	0	Japan
10	Stuart, MJ	19	281	14.79	9	4	0.69	0	0	USA

DF = dominance factor.

### 3.9. Publication trend of 3 important meniscus repair techniques

**Figure 8.**
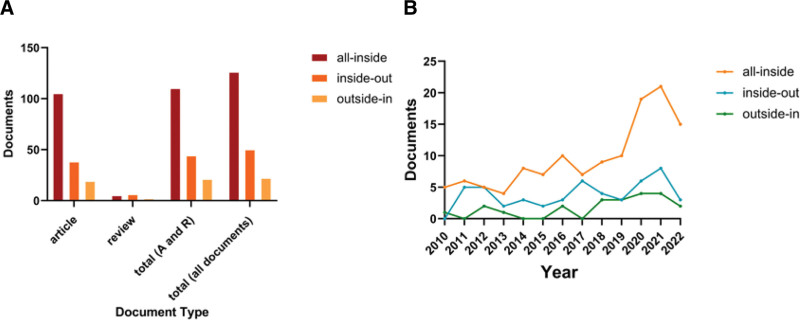
Visualization of the publication trends of the main 3 meniscus suture techniques. (A) The number of articles, reviews, articles and reviews and all types of documents of 3 suture techniques were shown respectively. (B) Distribution of publications on inside-out, outside-in, and all-inside techniques between 2010 and 2022. (A) and (B) were both made by GraphPad Prism.

There are 3 important techniques for meniscus repair: all-inside, outside-in and inside-out techniques. 950 retrieved documents were screened manually with titles containing “all-inside,” “outside-in” or “inside-out.” From 2010 to 2022, the number of documents with “all-inside” in titles was distinctly more than the other 2, especially in article (Fig. 8A). The distribution of publications showed that the number of publications on all-inside suture has increased obviously, indicating that researchers paid more attention to this technique. However, the annual publications on inside-out and outside-in repair techniques did not rise and remained at a low level (Fig. 8B).

## 4. Discussion

This study performed a bibliometric analysis of published papers concerning the meniscus suture from 2010 to 2022. By analyzing the all retrieved 950 papers, the research of meniscus suture was rising since 2010. The publication downward trend after 2020 was presumed to be due to the pandemic of coronavirus disease 2019. All-inside technique was gradually becoming a mainstream treatment for meniscal tears and the meniscus healing and meniscus root tears have been the research hotspots recently.

As one of the most common articular injuries, meniscal tears were traditionally treated by meniscectomy, however, it severely resulted in the occurrence and development of KOA.^[17]^ Therefore, meniscus suture was gradually developed for meniscus tears to preserve the functional integrity of load-bearing, shock absorption, and knee stability.^[24,34]^ The first meniscal suture was performed by Annandale in 1883, and great evolutions have been made in surgical treatments of meniscal tears with the improvement of arthroscopy and suturing devices.^[35–37]^

Currently, the upward trend of meniscus suture-related publications was performed over the past decade. In this study, a total of 950 publications from WOSCC met the search criteria. The number of annual publications has quadrupled in the past 12 years, indicating the increased attention of researchers worldwide to the meniscus suture. The United States, Japan, China and South Korea led the research field of meniscus suturing. The United States, as the most predominant contributor, published 36.63% of the papers with 348 papers and 7963 citations, which was far ahead of subsequent countries. China is growing to the newly promising contributor to further investigate the clinical outcomes using different suturing techniques based on the huge population. Additionally, Robert F LaPrade, Takayuki Furumatsu, and Toshifumi Ozaki are most-cited authors in this field. Moreover, the 177 journals were divided into core zone (n = 4), middle zone (n = 15) and minor zone (n = 158). The 4 journals of core zone are *American Journal of Sports Medicine, Arthroscopy-The Journal of Arthroscopic and Related Surgery, Knee Surgery Sports Traumatology Arthroscopy* and *Arthroscopy Techniques*. And 40.95% of articles published in these 4 journals.

In clinical practice, the meniscus suturing is mainly grouped into inside-out, outside-in, and all-inside techniques.^[24,34]^ Notably, although the inside-out meniscal suturing still remains as the referring standard for the treatment of most of types of meniscus tears,^[37]^ the publications concerning the all-inside technique were significantly increased in recent years (Fig. 8). Initially, the inside-out suturing is the first technique under the arthroscopic visualization in cost-effective and versatile fashion, and thus it is regarded as the gold standard for meniscal suturing.^[37]^ But the inside-out technique is usually concomitant with neurovascular injuries, increased operation time, and post-operative stiffness.^[16,38]^ Therefore, with the development of meniscus suturing devices, the all-inside suturing has emerged and evolved as the mainstream surgical strategy to repair torn menisci. The all-inside technique has advantages of better restoration of tissue continuity, preferable stability of knee joint and more independent meniscus movement, and it could restore the knee contact area close to its native state in more degrees of flexion.^[16,39]^ The all-inside technique showed comparable clinical outcomes versus inside-out technique as well.^[16,37,40]^ A meta-analysis performed by Elmallah et al^[41]^ found that all-inside and inside-out techniques had comparable healing rates, complication rates and outcome scores. In a systematic review, it was reported that there was no difference in clinical outcomes between all-inside and inside-out techniques.^[42]^ Although there is an increasing number of studies to compare the long-term clinical outcomes between different suturing methods, the inclusion and exclusion criteria and surgical details need to be clarified and the prognostic indicators of clinical outcomes need to be standardized. And more cohort studies and randomized controlled trials may further shed light on the advantages and disadvantages of these suturing techniques. In addition, the number of publications concerning outside-in technique unsurprisingly remained at a relatively low level, because it was primarily limited in the repair of the anterior horn and middle segment of meniscal tears.^[43]^

The “meniscus healing,” “meniscus root tear” may be the research hotspots in recent years through keywords analysis and will last in the future. In Figure 4B, the yellow nodes were the keywords that appeared more frequently after 2019. The keywords “platelet-rich plasma,” “magnetic resonance imaging,” “second-look arthroscopy” and “meniscal healing” all belonged to the research highlight of “meniscus healing.” Similarly, the keywords “root repair,” “posterior root tear,” “pullout repair,” “meniscal root repair” and “medial meniscus posterior root” could be seen as the embodiments of the research hotspot of “meniscus root tear.” With regard to postoperative meniscus healing, meniscus suture still had a higher incidence of failure and retear after initial surgery, which was indirectly reflected by increased relevant publications. In a systematic review, it was reported that the failure rate of meniscus suture remained at 19% in long-term studies.^[44]^ Although the cause of the failure is seldomly documented, the low healing capacity of meniscus may be one of the main causes, especially the inner avascular region (white-white zone).^[24,45]^ Therefore, many novel techniques were implemented to stimulate meniscus regeneration and to increase the healing capacity after meniscus suture, such as platelet-rich plasma (PRP) and autologous growth factors.^[46,47]^ Although meniscus root tears account for only 10% to 20% of all meniscus tears, “meniscus root tear” was still the research highlight in the field of meniscus suture due to subsequent impairment of meniscus and a study conducted by Allaire demonstrated that medial meniscal root tears are equivalent to subtotal medial meniscectomy.^[13,48]^ Furthermore, meniscus repairs resulted better outcomes in meniscus root tears than other treatments.^[48]^ Most of patients treated by either meniscectomy (99.3%) or non-operative treatments (95.1%) inevitably progressed in OA over 10 years, while the patients of meniscus repairs only accounted 53.0%.^[49]^ Besides, it has been proven that meniscus root attachments were crucial when meniscus performed its main function (shock absorption), as the root attachments could anchor the meniscus and prevent extrusion during tibiofemoral joint loading.^[50]^ Currently, the transtibial pullout repair was regarded as the gold standard for root tears due to its restoration for the normal contact mechanics.^[48,51]^

This study has several strengths. Firstly, the bibliometrics analysis of meniscus suture was performed to visualize author and institution collaborations, important journals and countries, and publication trends of different meniscal suture techniques. And a variety of tools, VOSviewer, GraphPad Prism 9 and R-bibliometrix, were utilized to summarize the research status based on present studies and to indicate the research hotspots and trends in a visualized fashion compared with traditional reviews. Secondly, WOSCC database served as the main source, providing a relatively comprehensive and well-defined information of relevant studies. The present study also has some limitations. First, this study only included the publication data from the WOSCC and all of studies were searched based on the title. Therefore, a few studies have been probably missed. But WOSCC is the most common and biggest database that contains the majority of publications, and the retrieval strategy ensured to cover almost all meniscus suturing-related studies. Second, the data was extracted from bibliometrics tools to provide visualized information, and thus the manual correction may be needed to improve the quality of raw data.

## 5. Conclusion

The number of publications concerning the meniscus suture increased year by year. The United States was the largest contributor to the field of meniscus suturing, followed by Japan, China and South Korea. And Robert F LaPrade was the most-cited author, and the most influential journal is *American Journal of Sports Medicine*. Although inside-out technique is still the gold standard for meniscus tears, all-inside technique is gradually becoming the mainstream suturing methods. The meniscus healing and meniscus root tears are the potential research highlights.

## Author contributions

**Conceptualization:** Wenhao Lu, Linyuan Pan.

**Data curation:** Xu Liu, Michael Opoku.

**Formal analysis:** Di Liu.

**Investigation:** Wenfeng Xiao, Heyuan Zhu.

**Project administration:** Yusheng Li.

**Resources:** Michael Opoku.

**Software:** Xu Liu, Di Liu.

**Supervision:** Wenfeng Xiao, Heyuan Zhu.

**Visualization:** Xu Liu, Wenhao Lu, Linyuan Pan.

**Writing – original draft:** Xu Liu.

**Writing – review & editing:** Xu Liu, Di Liu
